# Mechanisms of Innate Lymphoid Cell and Natural Killer T Cell Activation during Mucosal Inflammation

**DOI:** 10.1155/2014/546596

**Published:** 2014-05-28

**Authors:** David Nau, Nora Altmayer, Jochen Mattner

**Affiliations:** ^1^Mikrobiologisches Institut-Klinische Mikrobiologie, Immunologie und Hygiene, Universitätsklinikum Erlangen, Friedrich-Alexander Universität Erlangen-Nürnberg, Wasserturmstraße 3/5, 91054 Erlangen, Germany; ^2^Division of Immunobiology, Cincinnati Children's Hospital, Cincinnati, OH 45229, USA

## Abstract

Mucosal surfaces in the airways and the gastrointestinal tract are critical for the interactions of the host with its environment. Due to their abundance at mucosal tissue sites and their powerful immunomodulatory capacities, the role of innate lymphoid cells (ILCs) and natural killer T (NKT) cells in the maintenance of mucosal tolerance has recently moved into the focus of attention. While NKT cells as well as ILCs utilize distinct transcription factors for their development and lineage diversification, both cell populations can be further divided into three polarized subpopulations reflecting the distinction into Th1, Th2, and Th17 cells in the adaptive immune system. While bystander activation through cytokines mediates the induction of ILC and NKT cell responses, NKT cells become activated also through the engagement of their canonical T cell receptors (TCRs) by (glyco)lipid antigens (cognate recognition) presented by the atypical MHC I like molecule CD1d on antigen presenting cells (APCs). As both innate lymphocyte populations influence inflammatory responses due to the explosive release of copious amounts of different cytokines, they might represent interesting targets for clinical intervention. Thus, we will provide an outlook on pathways that might be interesting to evaluate in this context.

## 1. Mucosal Surfaces

Mucosal surfaces represent large areas in which some key interactions of the host with its environment occur [1]. The airway epithelium, for example, is critical for the O_2_-CO_2_ gas exchange, while the intestinal epithelium is required for the absorption of essential nutrients and vitamins. In contrast, however, intestinal as well as airway epithelial cells provide also a physiological barrier function against harmful substances and microbial pathogens [2, 3].

**Figure 1:**
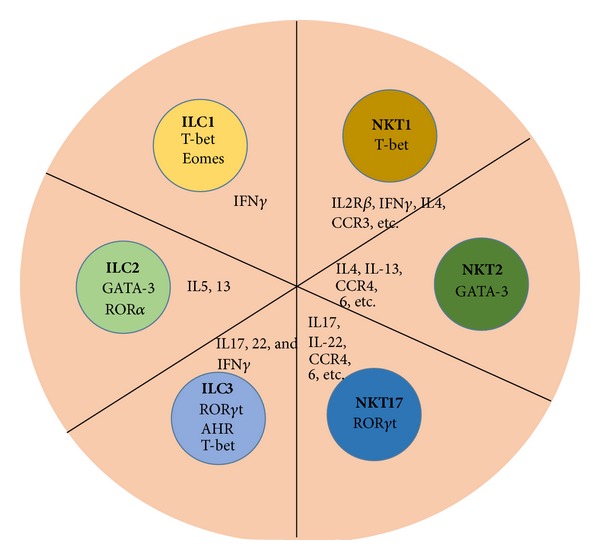
Conserved lineage diversification in ILCs and NKT cells. NKT cells as well as ILCs can be divided into three separate sublineages that resemble Th1, Th2, and Th17 subsets in conventional CD4-positive T cells. Group 1 ILCs are characterized by the expression of either T-bet (ILC1) or T-bet and Eomes (NK cells). Group 2 ILCs are defined by the expression of ROR*α* and GATA3 as well as their ability to release Th2 cytokines. Three sublineages are summarized under the umbrella of group 3 ILCs. While NK cell receptor-negative ILC3s only depend on ROR*γ*t, lymphoid tissue-inducer (LTi) cells require the aryl hydrocarbon receptor (Ahr) in addition to ROR*γ*t. NK cell receptor-expressing ILC3s depend on three transcription factors, AhR, ROR*γ*t and T-bet.

In order to distinguish between harmless and pathogenic triggers and antigens [4, 5], the mucosal immune system has evolved specific strategies distinct from its systemic counterpart to maintain tolerance on the one hand and also to mount protective responses on the other [6]. Mucosal epithelial cells, for example, play a key role in host defense by providing both a physical barrier and innate defense mechanisms such as the release of defensins. Dendritic cells (DCs) scavenge the mucosal surfaces for microbial antigens, promote T cell independent IgA responses by B cells, and also shape the adaptive T cell response within the mucosa and associated lymphoid tissues [7]. As the cytokine milieu is important for the differentiation of tolerance or inflammation, the abundant presence of specified innate immune cells such as innate lymphoid cells (ILCs) and natural killer T (NKT) cells [8–12] that release copious amounts of different cytokines and chemokines upon activation is critical for the generation of regulatory tolerogenic or inflammatory Th1-, Th2-, and Th17-dominated immune responses. Thus, while both cell populations are critical for the maintenance of mucosal tolerance, ILCs as well as NKT cells have been implicated in the induction of inflammatory and autoimmune diseases [13, 14]. These two cell populations share conserved polarized effector programs (Figure 1), although the molecular pathways involved in this sublineage differentiation must be also divergent as NKT cells, but not ILCs, for example, express a TCR. Here, we will discuss the mechanisms of activation as well as the capacity of these two cell subsets to modulate inflammatory immune response due to the release of copious amounts of different cytokines.

## 2. Development and Differentiation of Innate Lymphoid Cells (ILCs)

Innate lymphoid cells (ILCs) form a group of developmentally related cells that are characterized through their lymphoid morphology and the absence of RAG-dependent antigen receptors as well as of myeloid and dendritic cell phenotypic markers [15]. Based on functional criteria, cytokine polarization, and transcription factor expression, the six distinct members of the ILC family identified so far can be categorized into groups 1, 2, and 3 ILCs (ILC1, ILC2, and ILC3) [15] (Figure 1). Although the transcriptional repressor Id2 as well as cytokine signaling through the *γ*_3_ chain of the IL-2 receptor and the IL-7 receptor are required for the development and/or the maintenance of all of these ILC subsets [16, 17], a common precursor cell despite being widely assumed has not been identified so far [14]. With the exception of natural killer (NK) cells that belong to the ILC1 group and ILC2 that both develop in the bone marrow [18–20], the site of generation for the other ILC subsets has not been identified so far.

Although ILCs share developmental similarities, the ILC sublineages express specific transcription factors that drive the generation of each subset individually [21–29]. Those include the signature transcription factors T-bet, GATA-3, and ROR*γ*t that are also detected in polarized NKT cell sublineages (Figure 1) and differentiated conventional CD4-positive T helper cell subsets [30–34]. While group 1 ILCs are characterized by the production of IFN-*γ* and the expression of T-bet (ILC1) or T-bet and Eomes (NK cells), group 2 ILCs are defined by the expression of ROR*α* and GATA3 as well as their ability to release Th2 cytokines. In contrast, three sublineages are summarized under the umbrella of group 3 ILCs. While all group 3 ILCs require ROR*γ*t for their development and function and produce IL-17 and IL-22, they are on the one hand distinguished by the expression of NK cell receptors. Lymphoid tissue-inducer (LTi) cells that are essential for the formation of lymph nodes during embryogenesis require the aryl hydrocarbon receptor (Ahr) in addition. In contrast, while NK cell receptor-negative ILC3s only depend on ROR*γ*t, NK cell receptor-expressing ILC3s require in addition to the AhR also T-bet [15]. However, at least some of the processes underlying this sublineage diversification of ILCs must be different from conventional T cells and NKT cells despite the similar polarization of cytokine responses, as cognate antigen recognition does not occur in ILCs. The exact molecular mechanisms underlying the generation of the respective ILC subsets and the divergent as well as convergent pathways with respect to conventional T cells and NKT cells need to be defined in the future.

## 3. Selection of NKT Cells

NKT cells recognize different (glyco)lipid antigens [35] presented by the atypical MHC I like molecule CD1d on antigen-presenting cells (APCs) [36–38]. While the presentation of endogenous (glyco)lipids is critical for the selection of NKT cells in the thymus [39], NKT cells can survive in the periphery without the presence of CD1d [40]. Although NKT and T cells share similar signaling pathways, the transcription factors driving the development of one or the other lineage are different [41, 42]. Recently, the promyelocytic leukemia zinc finger PLZF has been defined as the lineage defining transcription factor in NKT cells [43–45]. While the thymic development of NKT cells has been divided into different stages that are characterized by the expression of distinct surface markers and transcription factors, this sequential lineage developmental paradigm for NKT cells was recently challenged by a model describing lineage diversification of NKT cells and simultaneous differentiation into Th1-, Th2-, and Th17-polarized subsets [46]. These polarized sublineages are characterized by the expression of the transcription factors T-bet, GATA-3, and ROR*γ*t [45], similarly as their conventional CD4-positive T helper cell counterparts and ILCs (Figure 1). Unlike conventional T cells, however, NKT cells do not require IRF-4 for the production of IL-17 and IL-22 [47]. Despite these distinct discrepancies in the polarization between NKT cells and conventional Th17 cells, the conserved expression pattern of ROR*γ*t, T-bet, and GATA-3 in ILCs, NKT cells, and conventional T cells suggest some similarities in the differentiation and cytokine-polarization of all three lineages. Nonetheless, the (molecular) mechanisms underlying this trifurcation of NKT cells are unknown and warrant further investigation.

## 4. Activation of ILCs and NKT Cells in the Periphery

Similarly like NKT cells, ILCs release copious amounts of cytokines upon bystander activation by distinct soluble factors. Those include different Th1, Th2, and Th17 cytokines and also regulatory cytokines such as IL-10, IL-2, or TGF*β*. Commensal as well as pathogenic microbes shape thereby the ILC response dependent on the cytokine profile they elicit on intestinal and myeloid cells [48, 49]. While NKT cells similarly like ILCs respond to IL-12, IL-18, IL-1, IL-23, and IL-33 [14, 50], only NKT cells, for example, respond to type 1 interferon [51]. Furthermore, the combination of IL-6 and TGF*β* decreased the CD3/CD28-mediated production of IL-22, but not of IL-17 [52]. DC-derived IL-1 and IL-23 were crucial for the IL-17-production of NKT cells in peripheral lymph nodes [53]. In contrast to NKT cells that can produce Th1 and Th2 simultaneously [54], ILC populations appear to be less plastic and exhibit either Th1-, Th2-, or Th17-restricted cytokine profiles [14, 15]. Furthermore, NKT cells can be also activated through the recognition of (glyco)lipid antigens (cognate activation) by their TCRs. Although NKT cells respond in the periphery to endogenous mammalian and exogenous microbial antigens, CD1d-presented signals are not required for the survival of peripheral NKT cells [55]. Thus, similarly like innate-like *γ*/*δ* T cells, which only need a strong TCR signal for proper development in the thymus [55], NKT cells might not require TCR signals for tissue surveillance in the periphery. However, there exist several microbes in which (glyco)lipid antigens have been detected [56–61] that can activate NKT cells directly through their TCR. Those TCR-activated NKT cells might shape the cellular network different than NKT cells activated through bystander activation by cytokines. The rapid arrest of NKT cells crawling through the liver sinusoids upon (glyco)lipid antigen encounter [62] might reflect one example of altered NKT cells behavior that is not observed when NKT cells react to soluble factors like IL-18 [63]. In addition, NKT cells become anergic for several weeks upon engagement of their TCRs [64]. In contrast, there exist no reports about ILC exhaustion and ILC might continuously release cytokines as long as their activating cytokine receptors such as IL-23R are expressed.

## 5. Role of ILCs and NKT Cells in Colitis and Asthma

Due to their potent immunomodulatory functions and abundance in mucosal tissues, ILCs and NKT cells have been associated with the disruption of mucosal homeostasis in the intestines and airways. Subsets of ILCs, for example, have been implicated in the pathogenesis of colitis [65] and airway inflammation [66–69] due to disturbances in the homeostasis of ILC subsets. While ILC3 cells protected from intestinal pathology, ILC1 subsets promoted mucosal damage. In contrast, predominantly ILC2 subsets were suspected to enhance epithelial damage in the airways due to the release of Th2 cytokines and the augmentation of adaptive Th2 responses. ILC-derived IL-9, a pleiotropic cytokine expressed at elevated levels in the lungs of asthmatic patients [70, 71], is thereby critical for the regulation of the Th2-cytokine release. In addition to the apparent pathogenic role of ILC2 cells which release predominantly Th2 cytokines, IL-17 produced by ILC3 subsets promotes airway hypersensitivity, particularly in the context of obesity [72]. Similarly, NKT cells contribute to the induction of pathology in mucosal tissues of the airways and the gastrointestinal tract. Thus, as Th2-polarized NKT cells are predominantly found in the lungs, they accelerate and worsen asthmatic disease [73, 74]. Comparable to the pathogenic role of ILC2s, NKT cells drive allergic inflammatory reactions through the production of type 2 cytokines and the recruitment and degranulation of mast cells and eosinophils [75, 76]. Similar pathogenic mechanisms might apply for the recruitment of eosinophils by NKT cells at other mucosal tissue sites, such as the esophagus [77]. While NKT cells in asthmatic mouse models induced by OVA are activated preferentially due to bystander activation, (glyco)lipid antigens detected in the fungus* Aspergillus fumigatus* or in cypress pollen drive cognate NKT cell activation and subsequently allergic airway inflammation [78, 79]. The nature of the (glyco)lipid engaging the NKT cell TCR might be thereby critical for the outcome of disease, as the application of alpha-galactosylceramide (*α*-GalCer), the prototypical NKT cell ligand, has been also associated with suppression of asthmatic immune reactions [80, 81]. However, particularly ILC2s produce also other cytokines, such as IL-25 and IL-33, that are critical for the maintenance of mucosal tolerance [82, 83] as well as the TNF family cytokine TLA-1 that promotes pathology [84]. Although the cognate recognition of the prototypical NKT cell ligand *α*-GalCer promoted pathogenic NKT cell responses dependent on the genetic background of the mice and the diseases investigated [85, 86], there exists also evidence for strong inflammatory effects on NKT cells through bystander activation, elicited, for example, through the application of poly-IC [87, 88]. On the other hand, the application of *α*-GalCer can also inhibit or ameliorate autoimmune diseases [89]. Although the processes underlying these inhibitory effects have not been completely resolved, cell-contact dependent interactions as well as the release of anti-inflammatory mediators have been suggested as protective mechanisms, which inhibit the expansion of autoreactive lymphocyte populations. However, while being the source of IL-22 that acts directly on epithelial cells [90], ILCs can also exhibit protective effects in mucosal tissues [91, 92]. A constitutive expression of IL-17 and IL-22 that is critical for the integrity of the intestinal mucosa and maintenance of epithelial homeostasis has been attributed to the ROR*γ*t-positive ILC3 subset [93, 94]. In contrast, Th17-cytokines, IL-13, and IFN-*γ* that are released by ILCs and NKT cells promote instead the pathogenesis of IBD [14, 95, 96]. In this context, there exists strong evidence that particularly IL-17-producing ILC3s as well as IL-23-reactive ILCs drive tissue pathology in IBD [97–100]. Thereby the local cytokine [101] as well as the microbial milieu [102] plays a critical role. In accordance with these studies, the depletion of an IFN-*γ*- and IL-17-producing ILC3 subset inhibited the development of intestinal pathology in a* Helicobacter hepaticus*-induced colitis model [103]. Furthermore, patients suffering from inflammatory bowel disease (IBD) revealed enhanced numbers of ILCs expressing IL-17 and IL-22 [104]. Th2- and Th17- polarized NKT cells have been implicated in the pathogenesis of colitis as well [105, 106]. In contrast to ILCs, however, the presence of the intestinal microbiota protected from NKT cell-mediated pathogenic responses in colitis [107, 108].

## 6. Interactions of NKT Cells and ILCs

Although sparse up to now, there exists experimental evidence for direct interactions of ILCs and NKT cells. While being target cells for ILC-produced cytokines, NKT cells enhance airway hyperreactivity and trigger lung pathology [109]. IL-25 plays thereby a critical pathogenic role as it promotes the production of IL-13 by nuocytes and NKT cells [110]. Besides allergic lung diseases, one publication reported also a regulation of ILC responses through NKT cells during viral infection [111]. NKT cells as well as alveolar macrophages represented in this model a cellular source for IL-33, which promoted the production of IL-5 by ILC2 cells. While these studies highlight the interactions of these two powerful innate immune cell populations, further studies need to define whether certain signal patterns can polarize the ILC and NKT cell response in one or the other direction or whether there exists a hierarchy in the interactions of ILC and NKT cell responses. With respect to IBD, certainly also the interactions of distinct components of the intestinal flora with ILCs and NKT cells warrant further investigation.

## 7. Summary and Outlook

ILCs and NKT cells play a critical role in the maintenance of mucosal homeostasis. Not only the various pattern recognition and cytokine receptors engaged but also the different sublineages involved under steady state and pathogenic conditions might shape the subsequent immune response of the complex downstream cellular network and influence the generation of inflammatory versus tolerogenic reactions. Thus, due to their potent immunomodulatory properties and their broad activation during inflammation, autoimmune disease, or infection both cell populations represent not only attractive targets for clinical intervention in the mucosa, but also at other tissue sites such as the liver or spleen. As both cell populations are characterized through polarized effector programs and lineage diversification (Figure 1), which are also known in the adaptive arm of the immune system, distinct common pathways certainly exist. However, although these diversification processes appear to be conserved, the receptors and molecular mechanisms driving this diversification cannot all be shared, for example, due to the involvement of TCR versus cytokine signaling. Although bystander activation induces cytokine responses in both cell populations, interference with cytokine receptors that are abundantly expressed also on other cell populations might elicit too many unspecific side effects. Thus, in order to polarize immune responses, ILCs as well as NKT cell subset specific surface molecules need to be identified and can be specifically targeted.
